# *optGpSampler*: An Improved Tool for Uniformly Sampling the Solution-Space of Genome-Scale Metabolic Networks

**DOI:** 10.1371/journal.pone.0086587

**Published:** 2014-02-14

**Authors:** Wout Megchelenbrink, Martijn Huynen, Elena Marchiori

**Affiliations:** 1 Institute for Computing and Information Sciences (ICIS), Radboud University Nijmegen, Nijmegen, The Netherlands; 2 Centre for Molecular and Biomolecular Informatics (CMBI), Radboud University Nijmegen Medical Centre, Nijmegen, The Netherlands; 3 Centre for Systems Biology and Bioenergetics (CSBB), Radboud University Nijmegen Medical Centre, Nijmegen, The Netherlands; University of Glasgow, United Kingdom

## Abstract

Constraint-based models of metabolic networks are typically underdetermined, because they contain more reactions than metabolites. Therefore the solutions to this system do not consist of unique flux rates for each reaction, but rather a space of possible flux rates. By uniformly sampling this space, an estimated probability distribution for each reaction’s flux in the network can be obtained. However, sampling a high dimensional network is time-consuming. Furthermore, the constraints imposed on the network give rise to an irregularly shaped solution space. Therefore more tailored, efficient sampling methods are needed. We propose an efficient sampling algorithm (called *optGpSampler*), which implements the Artificial Centering Hit-and-Run algorithm in a different manner than the sampling algorithm implemented in the COBRA Toolbox for metabolic network analysis, here called *gpSampler*. Results of extensive experiments on different genome-scale metabolic networks show that *optGpSampler* is up to 40 times faster than *gpSampler*. Application of existing convergence diagnostics on small network reconstructions indicate that *optGpSampler* converges roughly ten times faster than *gpSampler* towards similar sampling distributions. For networks of higher dimension (i.e. containing more than 500 reactions), we observed significantly better convergence of *optGpSampler* and a large deviation between the samples generated by the two algorithms. **Availability:** *optGpSampler* for Matlab and Python is available for non-commercial use at: http://cs.ru.nl/~wmegchel/optGpSampler/.

## Introduction

Modelling metabolic networks helps to unravel the complex machinery of metabolism within the cell. A classic approach is to model the reaction pathways in a dynamic fashion, using detailed kinetic data. For genome-scale models, often involving hundreds or thousands of reactions and metabolites, it is experimentally prohibitive to obtain the kinetic parameters involved. A constraint-based approach has successfully been applied to model and address a wide range of biological questions in the absence of detailed kinetic data [1], [2]. By using a steady-state assumption, a first type of constraint dictates that all metabolite concentrations stay constant over time (mass-balance). A second type of constraint limits the flux rate for each reaction (flux-capacity and directionality). The relation between the 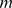 metabolites and 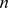 reactions is described in the 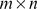 stoichiometric matrix 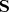. A positive stoichiometric coefficient 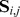 means that the metabolite 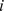 is produced by reaction 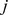 and a negative entry indicates that the metabolite is consumed in that reaction. At steady-state, the mass-balance and flux capacity constraints can be formulated as in eq. (1) and inequality (2) respectively.
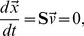
(1)

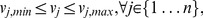
(2)


where 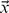 and 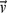 are vectors of metabolite concentrations and flux rates respectively. Each flux rate 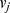 is bounded by inequality constraint (2). Although in some cases the bounds are known from experiments, for most reactions this is not the case and arbitrarily large values are used. Since the matrix 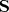 is a system of linear equations, the constraints in eq:mass-balance and inequality (2) form a bounded convex space [3], containing all possible values of 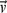. A major challenge is to characterize the biologically interesting flux distributions among all alternatives. Since the stoichiometric matrix is fixed, the remaining possibility is to add additional constraints or tighten the inequality bounds in (2). This can be done by incorporating measured flux data, which is often laborious, expensive or difficult to obtain, even for a small subset of all reactions.

Many constraint-based methods have been proposed to find flux distributions that are of biological interest. A successful approach is Flux Balance Analysis (FBA) [4], that introduces a biologically relevant objective function and uses linear programming to find a flux distribution that optimizes this objective. FBA proved to be especially useful for cell types with a well-defined objective function, such as maximum growth for unicellular species. Although FBA has successfully been applied to determine possible phenotypes and byproduct secretion in various experimental settings, it is often unable to determine the underlying (internal) flux states [5]. Furthermore, for many objective functions, a wide range of alternative optima exists [6].

In order to obtain an estimated probability distribution of attainable flux values for each reaction in the network, methods based on uniform sampling are used. A fast algorithm for this task is called *hit-and-run* (HR) [7]. HR collects samples by iteratively choosing a random direction and a random step size in that direction such that the next point also resides in the solution space. For the irregularly shaped solution spaces of metabolic networks the Artificial Centering Hit-and-Run (ACHR) algorithm [8] is better suited, because it is tailored to sample in the elongated directions of the solution space. Partly based on ACHR, the uniform random sampling procedure known as *gpSampler* is often used to sample metabolic networks and implemented in the COnstrained Based Reconstruction and Analysis (COBRA) Toolbox [9]. Although these algorithms are often referred to as uniform random samplers, their convergence behaviour in the context of genome-scale metabolic networks has not yet been thoroughly investigated. Besides its irregular shape, the solution space of genome-scale metabolic networks are often high-dimensional, containing hundreds to almost a thousand dimensions as in the human metabolic network reconstruction.

Therefore in this paper we investigate uniform random sampling in the context of metabolic networks. Our contributions are threefold: (1) we introduce an efficient and effective random sampling algorithm which combines the advantages of ACHR and *gpSampler*; (2) we propose a new measure to quantify the deviation between samples obtained from two independent sampling runs; (3) we perform a thorough analysis on five metabolic network models.

## Methods

### Uniform Random Sampling

One of the first attempts to sample the flux states in a metabolic network used a *rejection sampling* technique [10]. In rejection sampling, the space of interest is enclosed by a regular shape, such as a parallelepiped. Samples are drawn from the uniform distribution over the parallelepiped and rejected if they violate the constraints for the enclosed space of interest. The nice feature of rejection sampling is that the samples are uniformly distributed over the enclosed space. However, fitting a regular shape tightly around the space of interest is hard and often impossible. This means that in higher dimensions, the volume of the enclosing shape grows explosively compared to the volume of the shape of interest [7]. In this case, a very large fraction of the samples have to be rejected, making rejection sampling an inefficient method for genome-scale models.

**Figure 1:**
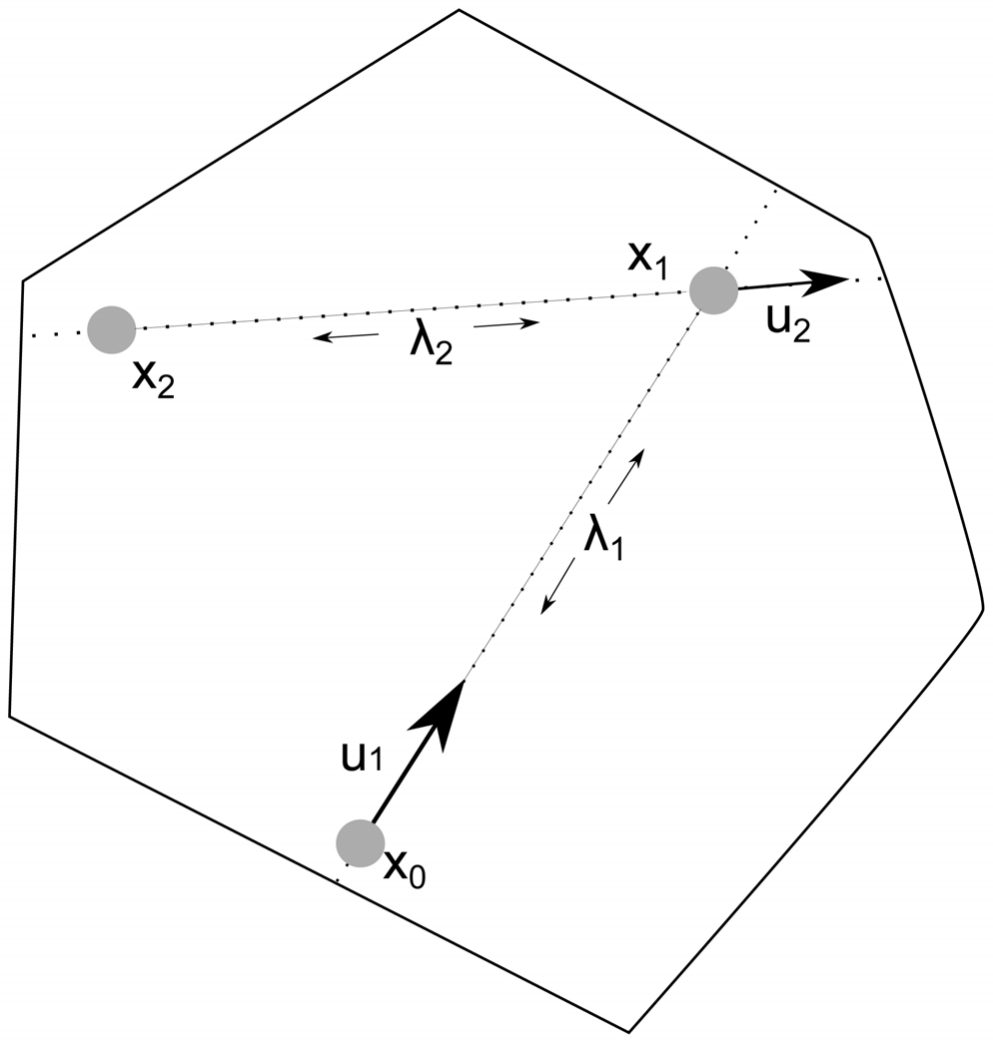
Illustration of hit-and-run. Hit-and-run starts at the point 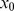 in the solution space 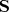. It chooses a random direction 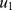 and determines the maximum distance it can travel forwards or backwards in that direction. A random step size 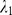 is chosen on the line 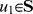. The next point 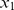 is obtained by travelling 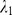 in the direction 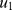. By iterating this process 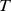 times, samples are obtained that are uniformly distributed in the space, when 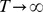.

The *hit-and-run* (HR) algorithm [7] mitigates this problem, because it samples directly from the solution space (see Fig. 1). Hit-and-run starts from a point 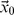 in the bounded space. It chooses an arbitrary direction 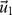 from the uniform distribution on the boundary 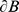 of the unit sphere in 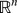. The distance from 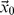 to the boundary of the solution space in the direction of 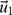 determines the maximum distance it can travel. An arbitrary step size 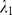 is selected in the (negative) direction of 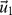, such that the sampler does not step out of the constrained space. The next point 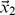 is determined by travelling distance 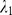 in the direction 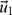. By iterating this process, HR generates a chain of consecutive sample points. The fact that HR uses only the current point to obtain the next sample makes it a Markov Chain Monte Carlo (MCMC) method [11], which has been shown to converge towards the target distribution (uniform in our setting).

In general, the constraints in metabolic network models lead to a convex space of irregular shape which is elongated for reactions whose flux rates are loosely constrained, and is narrow for tightly constrained fluxes. A consequence of this phenomenon is that many sample points are close to the boundary of the solution space. Since HR chooses a direction 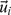 uniformly from all possible directions, this enforces the sampler to perform small steps, so the next generated point is close to the previous one. In practice this prevents the sampler to fully explore the rest of the solution space.

**Figure 2:**
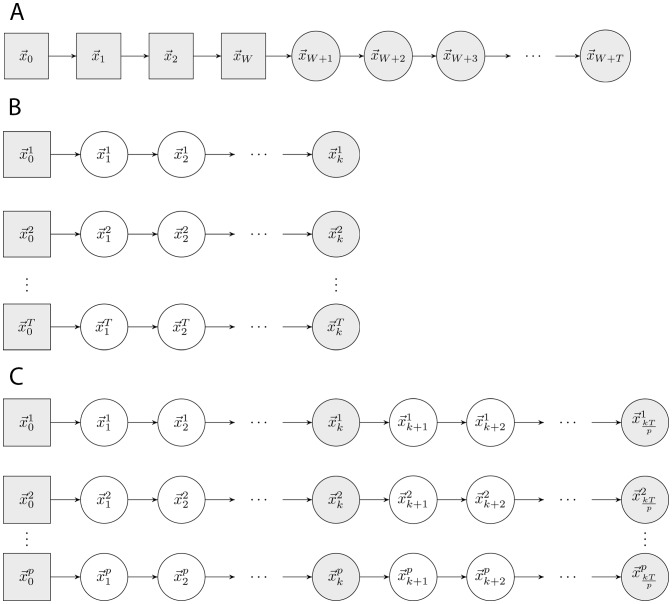
Conceptual difference between the samplers. Conceptual difference between (a) ACHR, (b) gpSampler and (c) optGpSampler. Warm-up points are depicted as gray rectangles, samples that are stored as gray circles. Uncoloured circles denote points that are visited by the sampler, but are not stored as a sample. a) The original ACHR algorithm starts at a point 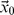 and iteratively moves to a next point 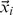 = 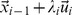. One chain is used, with step count 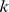 = 1. The chain contains 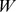 warm-up points and 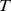 samples. b) *GpSampler* uses the linear programming procedure described in the main text to find 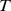 warm-up points. Then, each of the warm-up points is iteratively moved in the space in the same fashion as the ACHR algorithm in (a), leading to 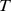 sampling chains. Each chain of length 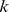 returns its end point as a sample. c) *OptGpSampler* obtains 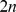 warm-up points. For each of the 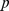 processors used, a warm-up point is chosen randomly as the initial point 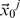, with 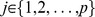. Starting from the warm-up points, new points are found in the same fashion as for the ACHR algorithm in (a), but now only every 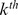 point is kept as a sample. Again, the result is 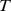 sample points, but now these have travelled 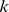 up to 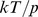 steps from a warm-up point. Compared to *gpSampler*, it uses less but much longer sampling chains.

ACHR [8] alleviates the problem of getting trapped in regions close to the boundary because it tries to sample in the elongated directions, thus making larger steps possible. It iteratively generates samples by using an ‘artificial’ centre of the space, which is empirically estimated at each iteration using the points sampled so far. ACHR consists of two phases: warm-up and (main) sampling. In the *warm-up* phase an arbitrary initial point 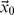 in the solution space is selected to generate a chain 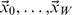 of 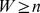 points. The requirement 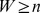 ensures that after the warm-up phase, the set of directions spans 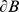 with probability one [8]. Then the main *sampling* phase starts from 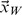. By iteratively updating the empirical centre and by using a direction from a randomly chosen previous sampled point to this centre, ACHR explores elongated directions of the space. Figure 2a illustrates the *warm-up* and the main *sampling* phase of ACHR.

The sequence of ACHR iterates is not a Markov chain, due to the dependence of directions on prior iterates. Thus the sequence of iterates is not guaranteed to converge to a uniform distribution [8].

ACHR is at the core of *gpSampler* [9], a popular sampling algorithm for metabolic network analysis. Figure 2b illustrates how *gpSampler* works. The highly irregular shape of the solution space of metabolic networks makes a uniform direction choice on 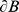 a poor choice. Therefore, in order to generate a number of 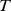 samples, *gpSampler*’s warm-up phase uses linear programming in a two parts procedure. In part (1) 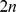 warm-up points are generated by consecutively minimizing and maximizing the flux rate of each reaction. In part (2) the remaining 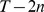 warm-up points are generated by assigning random weights to the fluxes that should be optimized. The optimal solutions (and thus the warm-up points) for a linear program reside on the boundary of the constrained space [12]. Often, this causes the allowed step sizes to become very small, which makes it hard to move away from the boundary. Therefore, *gpSampler* uses a linear transformation to ‘pull’ the warm-up points more into the interior of the solution space. Then, each moved warm-up point is used in the main sampling phase to generate 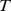 separate chains of length 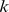. The user provided *step count* parameter 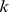 determines the number of ACHR iterates between the starting point 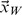 and the end point 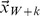 of each chain. Finally, the 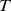 end points of the chains are returned as samples.

### optGpSampler

We propose to combine a part of the warm-up phase of *gpSampler* and ACHR. The resulting algorithm is called *optGpSampler* (see Table 1. Algorithm 1). First, 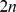 warm-up points are generated using part (1) of *gpSampler*’s warm-up procedure by successively minimizing and maximizing the flux through each reaction. We do not generate 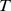 warm-up points as in *gpSampler* because running a linear program on a large network is much more time-consuming than random sampling. Moreover, although the weight vector for the linear program is randomly chosen, the constraints in eq. (1) and inequality (2) often lead to the same or a similar optimal solution. This could bias the starting points and directions choice of our sampler.

**Table 1:** Algorithm 1.

**Input:** 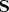 : the solution space; 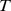 : sample count; 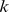 : step count, 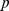 : number of processors;**output:** 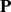 : sequence of 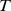 sampled points;/* Warm-up phase*/1 Generate 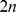 warm-up points as in part (1) of *gpSampler*’s warm-up phase;/* Sampling-phase*/2 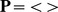 ;3 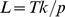 ;4 **for** 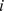 = 1 **to** 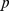 **do**5 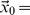 a point randomly chosen from the 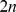 warm-up points;6 **for** 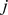 = 1 **to** 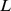 **do**7 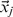 = point generated from 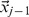 by performing one iterate of the ACHR sampling phase;8 **if** 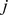 mod 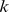 = = 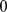 **then**9 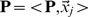 10 **end**11 **end**12 **end**

optGpSampler(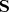, 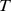, 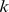, 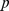).

The sampling phase of *optGpSampler* is similar to that of ACHR (see Fig. 2c), but only selects a sample at each 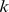 iterates. Furthermore, instead of generating one chain of consecutive sample points, *optGpSampler* exploits 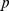 processors and generates 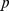 chains in parallel. In practice, the desired number of sample points 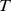 is much larger than the number of available processors 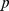. This makes the length of the chains generated by *optGpSampler* a factor of 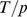 longer than those generated by *gpSampler*.

We implemented *optGpSampler* in C++ and used Armadillo [13], a fast linear algebra library. OpenMp [14] was utilized to start a separate chain on 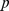 processor cores in parallel. Interfaces for Matlab and Python enable users to easily sample existing models with *optGpSampler* or integrate our sampler in new methods.

## Experiments

### Datasets

We benchmarked *gpSampler* and *optGpSampler* on five publicly available reconstructions of genome-scale metabolic networks [14]. All reactions and associated metabolites that could not carry a flux were removed from the models prior to the sampling. The network size remaining after this preprocessing step, i.e. the number of 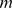 metabolites and 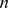 reactions is given between brackets. In ascending size order, we used E. coli central metabolism (68, 87), C. thermocellum iSR432 (288, 351), S. cerevesiae iND750 (479, 631), E. coli iAF1260 (1032, 1532) and H. sapiens recon 1 (1587, 2469).

### Evaluation

We assessed efficiency and quality performance of *gpSampler* and *optGpSampler*. Efficiency was measured using *runtime*. Quality performance in our context amounts to measure the capability of a sampler to effectively generate points uniformly distributed in the solution space. Since both *gpSampler* and *optGpSampler* have no theoretical convergence guarantees, we use two methods: empirical convergence diagnostics and a new method called xy-deviation.

### Empirical Convergence Diagnostics

Empirical convergence diagnostics are used to test whether the sampled distribution converges towards a stationary one. There is not always good agreement between different convergence diagnostic methods [16]. Therefore we use the following three convergence diagnostics tests: Gelman and Rubin [17], Geweke [18] and Heidelberger and Welch (HW) [19]. They are available in the Convergence Diagnosis and Output Analysis (CODA) Toolbox for MCMC [20].

The Gelman and Rubin test is multivariate: it returns a so called 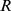 value (with 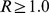). 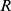 values smaller than 1.2 indicate convergence, with values closer to 1.0 indicating better results. The other two tests are univariate. The Geweke test returns a z-value for each reaction, with lower values indicating better convergence. The HW test returns whether a sample distribution for a given reaction converged (value = 1) or not (value = 0).

### xy-Deviation

In general, a larger step count gives a sample distribution that is closer to the target distribution, at the expense of a longer runtime. Therefore, we introduce a measure called 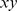-deviation that quantifies how samples generated by sampler *x* using a given step count 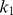 deviate from those generated by sampler *y* using a *much larger* step count 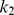. Specifically, a small deviation indicates that the sample distribution generated by sampler 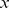 converged empirically to the target distribution of 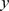.

Given samplers 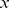 and 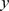, step counts 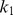 and 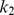, with 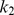 very large (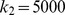 in our experiments), the number 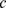 of runs, and the number 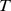 of samples, 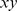-deviation is computed as follows.

1. Perform 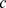 runs of sampler 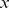 with step count 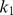, and 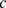 runs of sampler 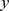 with step count 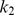, where each run generates 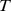 samples.
2. Sort the points in each run (e.g., in increasing order), producing 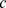 sorted chains of points for sampler 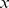 and 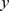: 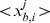, 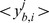, 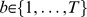, 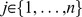, 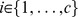.
3. For each reaction 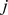, normalize the flux rates: we divide 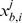 (resp. 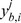) by the difference between the upper and lower bounds of 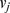:

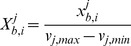
, 
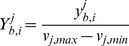
, 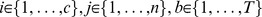.
4. Compute the mean chain of the 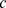 runs of 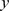:

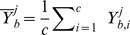
, 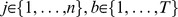.
5. Our assumption is that chains generated by sampler 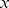 that converge will have small deviation from the ‘average-chain’ generated by 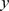. For each reaction 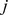, we measure such deviation by computing the mean absolute deviation over the chains generated by 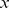 from 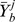: 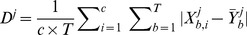, 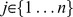.
  Our choice to use a mean deviation over the standard deviation is motivated by the fact that the mean deviation is more efficient than the standard deviation in the realistic situation where some of the measurements are in error, and more efficient for distributions other than perfect normal [21]. Note that the range of 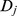 is [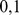], and can be expressed as a percentage, which makes it convenient to compare deviations across different reactions.
6. Then the *xy-deviation* over 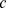 chains with respect to 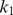 and 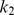 is defined as the average of the reaction deviations:

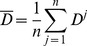
(3)Small values of *xy-deviation* indicate convergence of 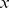 to 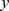. In the experiments we analysed self-deviation (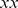-deviation) and cross-deviation (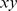-deviation, with 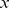 and 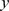 different samplers) for different values of 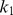.

## Results

We used the *gpSampler* implementation in the COBRA Toolbox. Results of extensive experiments are given in supplementary material. Tables S1–S5 in File S1 provide results for all the runtime experiments performed. Figures S1–S6 in File S1 provide the results of the convergence diagnostic tests and 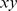-deviation. We visualized how a small or large 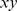-deviation translates to a similar or (highly) dissimilar sample distribution in figures S7–S11 in File S1.

### Efficiency

We sampled all networks four times using 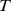 = 10, 50 and 100 thousand samples, with step count 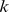 = 50. Both *gpSampler* and *optGpSampler* were executed in parallel mode on an AMD desktop computer using 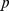 = 4 processor cores in parallel. Efficiency results, summarized in Table 2, show that *optGpSampler* is roughly 6 to 40 times faster than *gpSampler*.

**Table 2:** Runtimes for the networks analysed.

Networkname	m	n	N(S)	Time*Gp* (SD)	Time*optGp* (SD)
E. colicore	68	87	24	137.16(0.62)	3.63(0.05)
C. therm.iSR432	288	351	70	258.57(0.54)	10.20(0.05)
S. cerev.iND750	479	631	180	496.57(3.07)	21.81(0.04)
E. coliiAF1260	1032	1532	525	1474.01(6.78)	95.78(2.62)
H. sapiensrecon 1	1587	2469	932	2910.26(43.57)	349.05(0.48)

The number of metabolites and reactions is denoted by *m* and *n* respectively. The dimensionality of the nullspace of 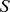, is given by *N(S)*. *Time gp (SD)* is the mean runtime (seconds) and standard deviation for sampling 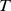 = 50.000 points using *gpSampler*. *Time optGp (SD)* denotes the same figures for *optGpSampler*.

Experiments were performed on a 16 GB RAM AMD Phenom desktop pc.

### Quality

To asses the quality of the results, we performed four independent runs with each sampler. Each run collected 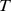 = 50.000 samples and was repeated for six different step counts (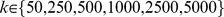).

We ran all convergence diagnostics with the default settings in the CODA package. The convergence tests results were averaged over the four runs. A univariate test for a sampler outputs a vector of length 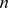. Significance of the difference between *gpSampler* and *optGpSampler* was assessed by applying the Wilcoxon signed-rank test to the corresponding vectors. The results for the Gelman and Rubin test indicated convergence for all experiments, in disagreement with results of the Geweke and HW tests.

The obtained chains were also used to compute the 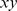-deviation for the above given step count 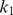, and 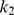 = 5000. All sample chains were compared by the average of the four chains obtained at the highest step count of 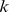 = 5000.

#### Small networks (less than 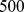 reactions)

On the *E. coli central metabolism* network, the Geweke test returned relatively low z-values for both *gpSampler* and *optGpSampler* (see figure S1 **in** File S1). Results of the HW test indicated that *optGpSampler* converged rapidly, at 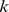 = 50 step count. Both the Geweke and HW tests showed that *gpSampler* needs a step count close to 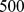 to converge. Results of xy-self-deviation demonstrate a large deviation of samples generated by *gpSampler* with a small step count from those generated with a higher step count. In general, results showed that *optGpSampler* with a low step count generates samples that are both close to those generated using a higher step count with the same sampler and to those generated by *gpSampler* with a higher step count.

**Figure 3:**
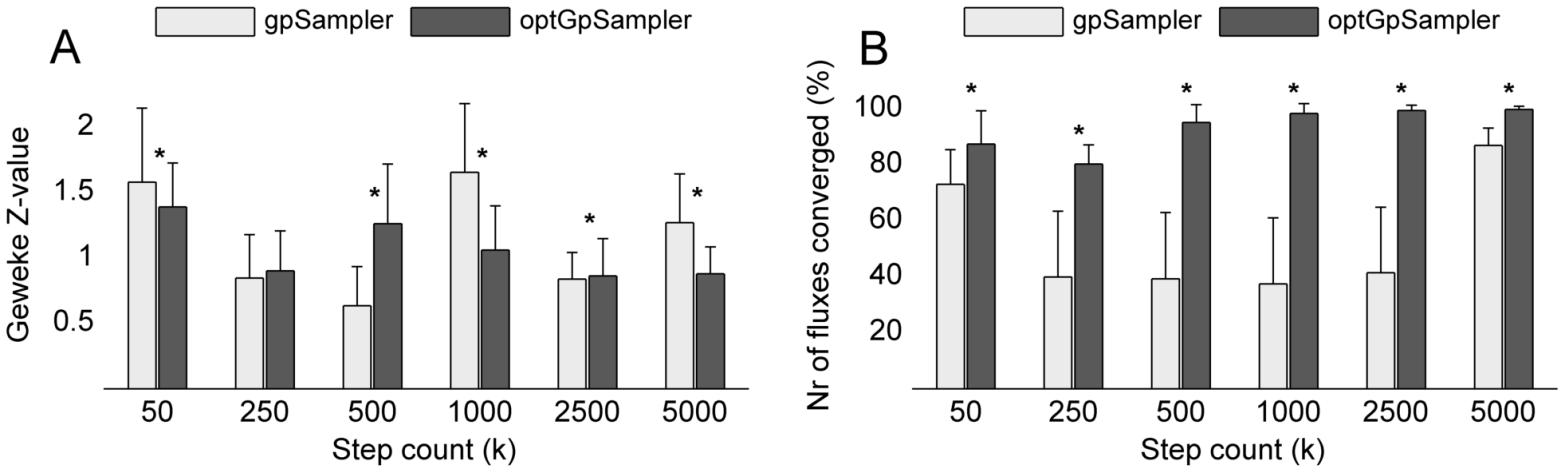
Empirical convergence for C. thermocellum iSR432. (a) Convergence according to the Geweke diagnostic. (b) Convergence according to the Heidelberger-Welch diagnostic. Convergence for *optGpSampler* is observed at approximately 500 steps. For *gpSampler*, both diagnostics only agree on convergence after 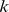 = 5000 steps. Notice the higher HW convergence fraction of the latter at 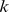 = 50 steps and at 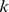 = 5000 steps compared to the steps in between.

**Figure 4:**
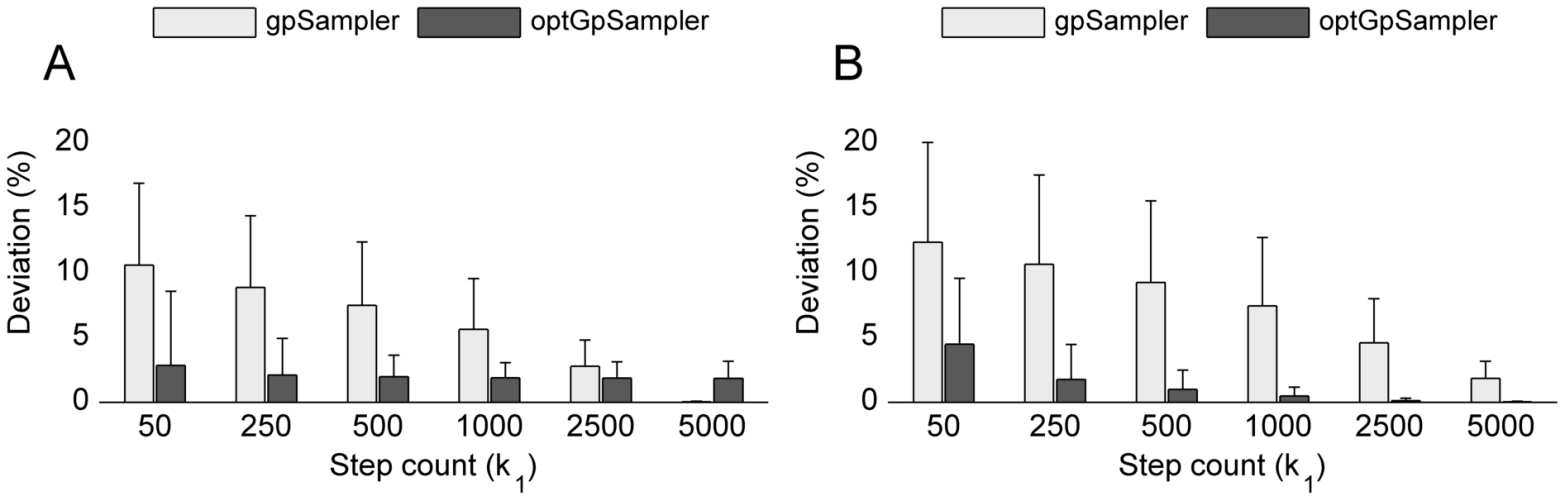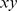-deviation for C. thermocellum iSR432. 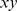
-deviation from samples obtained with sampler 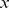 at step count 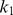 to sampler 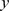 using 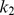 = 5000. (a) Deviation to samples obtained by 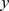 = *gpSampler*. (b) Deviation from samples obtained by 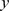 = *optGpSampler*. In both cases *optGpSampler* converges much faster to sampler 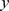.

On the *C. thermocellum iSR432* network, the HW test showed significantly higher convergence rates for *optGpSampler* (see Fig. 3). Both the Geweke and HW tests showed that *optGpSampler* converged at step counts bigger than 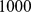. The situation for *gpSampler* is different: the values of the HW test dropped at step count 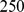 and then increased significantly when the step count reached 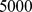. This indicates two distinct points of convergence, one at small step count values and one at large values. This behaviour can be explained by the way *gpSampler* collects its samples. It starts at a warm-up point and uses 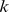 iterates of the ACHR algorithm to obtain a sample point. It repeats this process, each time starting from a different warm-up point (see Fig. 2b). These warm-up points turn out to be close to each other, as a consequence of the linear programming procedure used. Thus *gpSampler* often starts the sampling from the same area of the solutions space. In higher dimensions, a small step count can prevent *gpSampler* to ‘travel’ far from the warm-up points. In this case the small step count causes *gpSampler* to show a bias towards the regions close to the warm-up points and the sampling chain converges towards these regions. The large 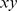-deviation (see Figure 4) shows that the samples collected are different from those sampled at a higher step count.

The results for *gpSampler* at large step count (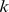 = 5000 steps) again indicates convergence. In this case the small 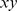-deviation indicates that an extended region is covered by the samples. Since *optGpSampler* does not restart at a warm-up point, it is better able to ‘escape’ from the regions near the warm-up points because it effectively uses much longer chains. Therefore, its convergence and 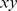-deviation results are better.

#### Large networks (at least 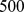 reactions)

**Figure 5:**
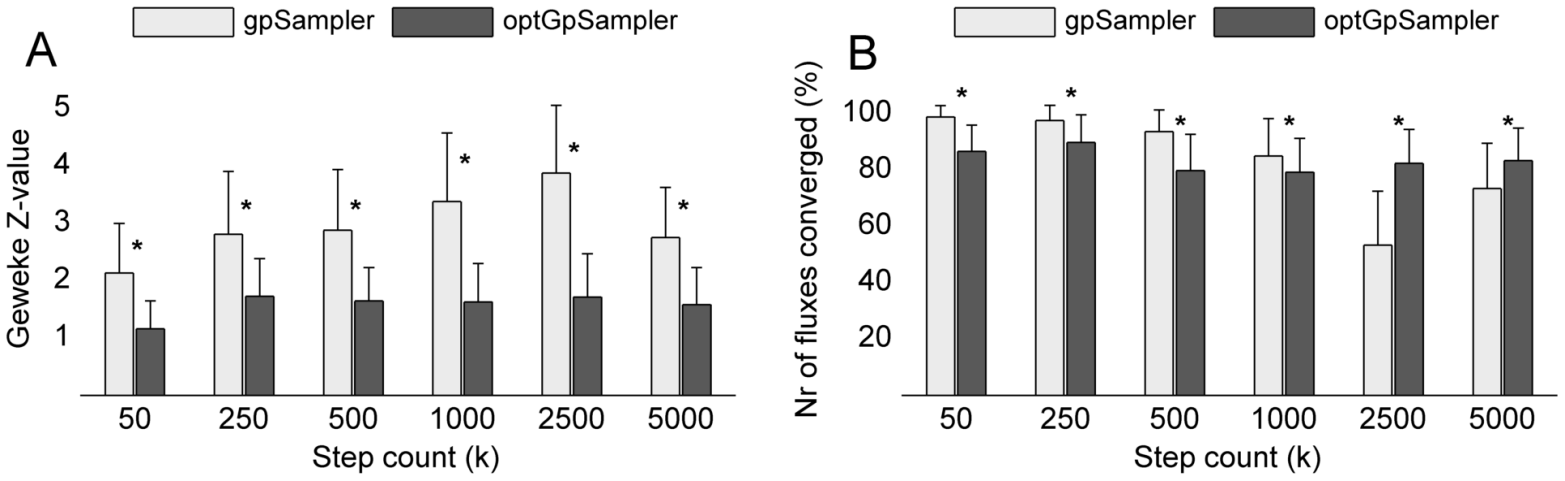
Empirical convergence for E. coli iAF1260. (a) Convergence according to the Geweke diagnostic. (b) Convergence according to the Heidelberger-Welch diagnostic. For both convergence tests, *gpSampler*’s performance deteriorates when the step count is increased up to 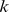 = 2500. Especially the good scores at low values of 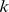 seem unrealistic and could indicate convergence towards a non-uniform distribution. Results for *optGpSampler* seem more stable and more reliable.

**Figure 6:**
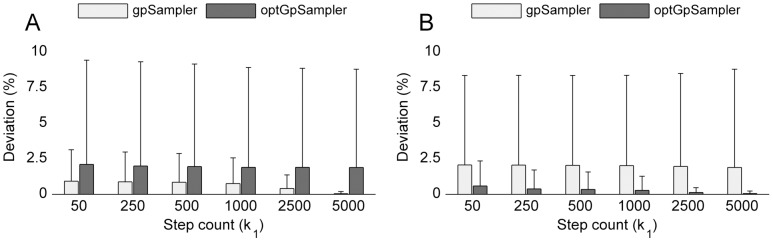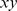-deviation for E. coli iAF1260 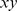-deviation from samples obtained with sampler 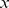 at step count 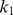 to sampler 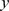 using 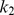 = 5000. (a) Deviation to samples obtained by 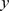 = *gpSampler*. (b) Deviation from samples obtained by 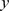 = *optGpSampler*. Self-deviation (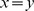) is small for both samplers, but there is a large cross-deviation (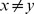). For this large network, the we do not observe convergence of *gpSampler* to the samples obtained by *optGpSampler* or vice versa as in Fig. 4.

The convergence and results for the *S. cerevisiae iND750* (see figure S3 **in** File S1) and especially the *E. coli iAF1260* network in Fig. 5 showed an even more surprising result. For these larger models, the convergence results for *gpSampler* declines when the step count is increased. For *optGpSampler*, we still observed an increasing convergence performance for the yeast network, although a much larger step count (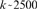) was required. The more stable results for the larger *E. coli iAF1260* network could be an effect of the minimum glucose setting of this network, which significantly reduces the attainable flux states. The 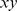-deviation results in Fig. 6 indicate that the samplers give completely different sampling results. The self-deviation shown in Fig. 6, reveals a small variability within the four independent runs for each sampler. This means that both samplers give relatively stable results, and thus that the deviation results observed between the samplers must be due to the difference between the algorithms.

Finally, the results for the human network reconstruction (see figures S5 and S6 in File S1) indicate that *gpSampler* converges already at low 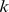. Although we believe that the sample distributions indeed converged in this case, it seems unlikely that they represent a uniformly distributed sample. First, the high dimensionality of almost a thousand and the declining results for the Geweke test make this unlikely. Next, the huge deviation with samples obtained by *optGpSampler* indicate a non-uniform distribution, especially since we saw that *optGpSampler* performs better on the smaller networks. The convergence results for *optGpSampler* seem more realistic, with a relatively large z-value and a HW test result that indicates that around 60% of the sampled flux distributions converged.

## Discussion

We proposed a new algorithm for uniform sampling of the steady-state solution space of metabolic networks. Our algorithm also implements ACHR, but in a different manner than the state-of-the-art sampling method for metabolic networks (*gpSampler*). We compared the runtimes with those of *gpSampler*, and showed its superior efficiency. We investigated empirical convergence using different diagnostics and showed faster convergence of *optGpSampler* on the two smaller networks studied. Moreover, by using the here introduced 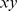-deviation measure, we compared the sampled distributions. For smaller networks, the samples obtained by *optGpSampler* using a small step count are close to those obtained with a high step count by both *optGpSampler* and *gpSampler*.

On three large networks the convergence performance of *gpSampler* diminishes when the step count increases. We hypothesized that the approach *gpSampler* takes by starting each sample chain at a warm-up point, together with the high dimensionality of the solution space, restrains its ability to move from the vicinity of these warm-up points. Because our method continues the ACHR procedure from the last collected point, it effectively uses much larger step counts. Therefore, *optGpSampler* is more likely to escape the regions near the warm-up points, leading to a better sampling result.

For the larger networks, results showed that the convergence observed at lower step counts does not reflect a convergence towards the target distribution, since the sample distributions deviate significantly from those generated using a large step count. Therefore, especially larger networks should be sampled with a high step count.

To the best of our knowledge there is no method to assess whether samples are truly uniformly random distributed in a convex space of unknown shape. Since the chains generated by ACHR are not Markov chains, asymptotic convergence guarantees also do not hold for both *gpSampler* and *optGpSampler*. Therefore convergence results should be interpreted with caution. The accelerated convergence of ACHR towards a uniform distribution was demonstrated by [8] for convex polytopes of known shape. However it remains uncertain to what extent the samples obtained by ACHR for the irregular solution space of metabolic networks are truly uniformly distributed. As expected, our experiments indicate that convergence results deteriorate when the dimensionality of the solution space increases, and that for the large genome-scale metabolic networks, using a large step count is advisable.

We envisage the provided implementation of *optGpSampler* will be beneficial to constraint-based metabolic network analysis, as it provides an efficient and versatile algorithm for sampling the irregular solution space of metabolic networks.

## Supporting Information

File S1
**Runtimes, convergence results and 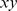-deviation for the other metabolic models considered.**
(PDF)

## References

[1] Kauffman KJ, Prakash P, Edwards JS (2003) Advances in ux balance analysis. Current opinion in biotechnology 14: 491–496. 14580578 10.1016/j.copbio.2003.08.001

[2] Raman K, Chandra N (2009) Flux balance analysis of biological systems: applications and challenges. Briefings in bioinformatics 10: 435–449. 19287049 10.1093/bib/bbp011

[3] Price ND, Reed JL, Papin JA, Wiback SJ, Palsson BO (2003) Network-based analysis of metabolic regulation in the human red blood cell. Journal of Theoretical Biology 225: 185–194. 14575652 10.1016/s0022-5193(03)00237-6

[4] Varma A, Palsson BO (1994) Metabolic ux balancing: Basic concepts, scientific and practical use. Bio/technology 12.

[5] Suthers PF, Burgard AP, Dasika MS, Nowroozi F, Van Dien S, et al (2007) Metabolic ux eluci- dation for large-scale models using 13c labeled isotopes. Metab Eng 9: 387–405. 17632026 10.1016/j.ymben.2007.05.005 PMC2121621

[6] Mahadevan R, Schilling C (2003) The effects of alternate optimal solutions in constraint-based genome-scale metabolic models. Metabolic engineering 5: 264–276. 14642354 10.1016/j.ymben.2003.09.002

[7] Smith RL (1984) Efficient monte carlo procedures for generating points uniformly distributed over bounded regions. Operations Research 32: 1296–1308.

[8] Kaufman DE, Smith RL (1998) Direction choice for accelerated convergence in hit-and-run sampling. Oper Res 46: 84–95.

[9] Schellenberger J, Palsson BØ (2009) Use of randomized sampling for analysis of metabolic networks. J Biol Chem 284: 5457–5461. 18940807 10.1074/jbc.R800048200

[10] Wiback SJ, Famili I, Greenberg HJ, Palsson BØ (2004) Monte carlo sampling can be used to determine the size and shape of the steady-state ux space. Journal of theoretical biology 228: 437–447. 15178193 10.1016/j.jtbi.2004.02.006

[11] Gilks WR, Richardson S, Spiegelhalter D (1995) Markov Chain Monte Carlo in Practice (Chapman & Hall/CRC Interdisciplinary Statistics). Chapman and Hall/CRC.

[12] Winston WL, Goldberg JB (2004) Operations research: applications and algorithms. Thomson/Brooks/Cole Belmont.

[13] Sanderson C (2010) Armadillo: An open source C++ linear algebra library for fast prototyping and computationally intensive experiments. Technical report, Nicta, St Lucia (Australia).

[14] OpenMP Architecture Review Board (2008) OpenMP application program interface version 3.0. Available: http://www.openmp.org/mp-documents/spec30.pdf.

[15] Schellenberger J, Park JO, Conrad TM, Palsson BØ (2010) Bigg: a biochemical genetic and genomic knowledgebase of large scale metabolic reconstructions. BMC bioinformatics 11: 213. 20426874 10.1186/1471-2105-11-213 PMC2874806

[16] Cowles MK, Carlin BP (1996) Markov chain monte carlo convergence diagnostics: A comparative review. Journal of the American Statistical Association 91: 883–904.

[17] Gelman A, Rubin DB (1992) Inference from iterative simulation using multiple sequences. Statistical Science 7: 457–472.

[18] Geweke J (1992) Evaluating the accuracy of sampling-based approaches to the calculation of posterior moments. In: IN BAYESIAN STATISTICS. University Press, pp. 169–193.

[19] Heidelberger P, Welch PD (1983) Simulation run length control in the presence of an initial transient. Operations Research 31: 1109–1144.

[20] Plummer M, Best N, Cowles K, Vines K (2006) CODA: Convergence diagnosis and output analysis for MCMC. R News 6: 7–11.

[21] Gorard S (2005) Revisiting a 90-year-old debate: The advantages of the mean deviation. British Journal of Educational Studies: 417–439.

