# Supplementary material for: optGpSampler: An Improved Tool for Uniformly Sampling the Solution-Space of Genome-Scale Metabolic Networks
Source: PLoS One. 2014 Feb 14;9(2):e86587. doi: 10.1371/journal.pone.0086587 (PMC3925089; doi:10.1371/journal.pone.0086587)
Supplement: File S1 — Runtimes, convergence results and -deviation for the other metabolic models considered. (PDF) [file pone.0086587.s001.pdf]

optGpSampler: an improved tool for uniformly sampling the solution-space of genome-scale metabolic networks
Supplementary Material

Wout Megchelenbrink, Martijn Huynen and Elena Marchiori

Contents

In this supplement we report results for all experiments conducted. Section 1 provides all *runtime* results for the Gurobi and IBM Ilog Cplex linear programming solvers that we used. In section 2 we show the convergence diagnostic and xy-deviation results for all metabolic networks considered. We illustrated how xy-deviation expresses the difference in sampled flux distribution in section 3.

1 Runtimes

The average runtimes and standard deviations over four independent runs are listed in Tables 1-5 for the five networks and the two linear programming solvers. In all cases *optGpSampler* is significantly faster than *gpSampler*. Runtimes increase with network size and grow roughly linear with the number of samples. The dimensionality of the solution space is denoted by $N(S)$. All result are obtained using step count k=50.

Table 1: **Runtimes for *E. coli core*, $N(S)$ = 24.**

Method	Samples ($\times 10^3$)	Runtime (Cplex)	Runtime (Gurobi)
gpSampler	10	26.46 (0.13)	27.98 (0.12)
	50	130.10 (0.37)	137.16 (0.62)
	100	263.51 (1.38)	277.88 (0.88)
optGpSampler	10	1.02 (0.01)	1.02 (0.01)
	50	3.70 (0.04)	3.63 (0.05)
	100	6.99 (0.06)	7.06 (0.09)

Table 2: **Runtimes for *C. thermocellum iSR432*, $N(S)$=70.**

Method	Samples ($\times 10^3$)	Runtime (Cplex)	Runtime (Gurobi)
gpSampler	10	47.01 (0.16)	52.12 (0.06)
	50	233.55 (1.44)	258.57 (0.54)
	100	470.86 (1.13)	521.61 (2.27)
optGpSampler	10	2.59 (0.03)	2.48 (0.01)
	50	10.24 (0.08)	10.20 (0.05)
	100	19.92 (0.19)	19.71 (0.12)

Table 3: **Runtimes for *S. cerevisiae iND750*,** $N(S)$ **= 180.**

Method	Samples ($\times 10^3$)	Runtime (Cplex)	Runtime (Gurobi)
gpSampler	10	85.48 (0.86)	98.21 (0.23)
	50	431.39 (1.82)	496.57 (3.07)
	100	877.68 (5.12)	1009.57 (10.83)
optGpSampler	10	5.31 (0.05)	5.22 (0.05)
	50	22.34 (1.50)	21.81 (0.04)
	100	41.98 (0.09)	42.40 (0.33)

Table 4: **Runtimes for *E. coli iAF1260*,** $N(S)$ **= 525.**

Method	Samples ($\times 10^3$)	Runtime (Cplex)	Runtime (Gurobi)
gpSampler	10	231.66 (0.89)	289.03 (0.72)
	50	1243.58 (15.64)	1474.01 (6.78)
	100	2476.75 (19.51)	3004.19 (9.21)
optGpSampler	10	24.70 (0.27)	24.07 (0.33)
	50	95.35 (1.71)	95.78 (2.62)
	100	184.91 (2.32)	185.53 (1.62)

Table 5: **Runtimes for *H. sapiens recon1*,** $N(S)$ **= 932.**

Method	Samples ($\times 10^3$)	Runtime (Cplex)	Runtime (Gurobi)
gpSampler	10	427.40 (6.11)	509.92 (4.23)
	50	2318.22 (4.95)	2910.26 (43.57)
	100	4920.02 (101.69)	5874.14 (60.66)
optGpSampler	10	92.46 (0.12)	84.92 (0.41)
	50	385.20 (0.70)	349.05 (0.48)
	100	746.74 (2.96)	678.09 (0.50)

2 Convergence and xy-deviation results

As mentioned in the main article, all results for the Gelman and Rubin test indicated convergence (R-score < 1.1). Since these results do not distinguish between *gpSampler* and *optGpSampler* and do not agree with the other two convergence test, we do not report further details for this test.

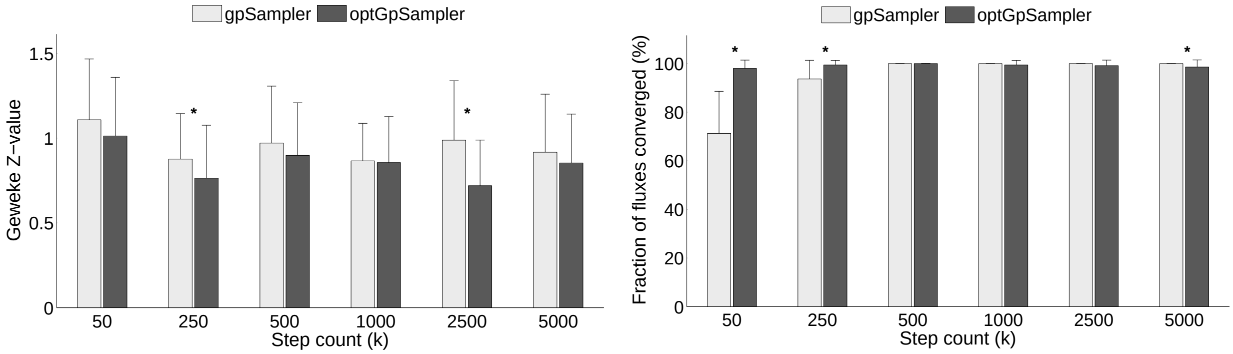

(a) Geweke diagnostic

(b) Heidelberger-Welch diagnostic

Figure 1: **Empirical convergence for E. coli central metabolism.** For this small network the samplers fully converge for both diagnostics. *OptGpSampler* converges around k=50 steps, whereas *gpSampler* converges near approximately k=500 steps.

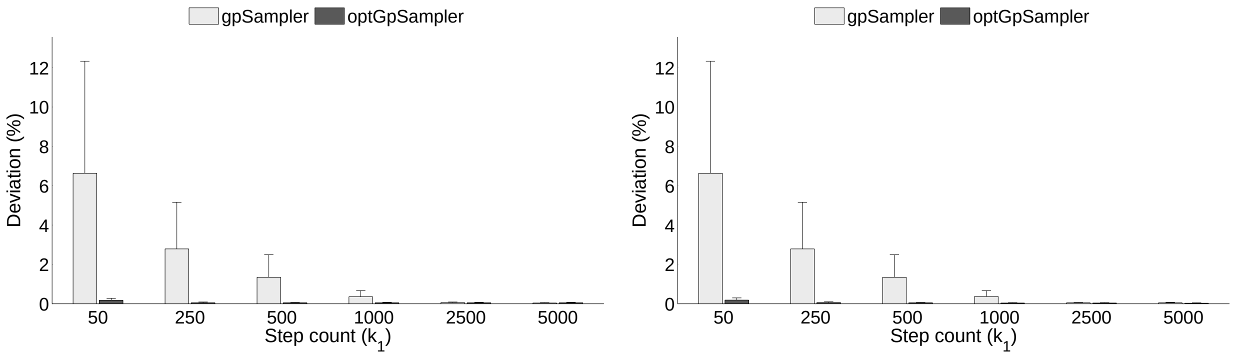

(a) y=gpSampler

(b) y=optGpSampler

Figure 2: xy**-deviation for E. coli central metabolism.**[1] xy-deviation of sampler x using step count k_1 to sampler y using k_2=5000. (a) y=*gpSampler*. (b) y=*optGpSampler*.
Notice the small deviation of *optGpSampler* from sampler y in both cases at all step counts.

[1]Although the figures look very similar, they are actually different.

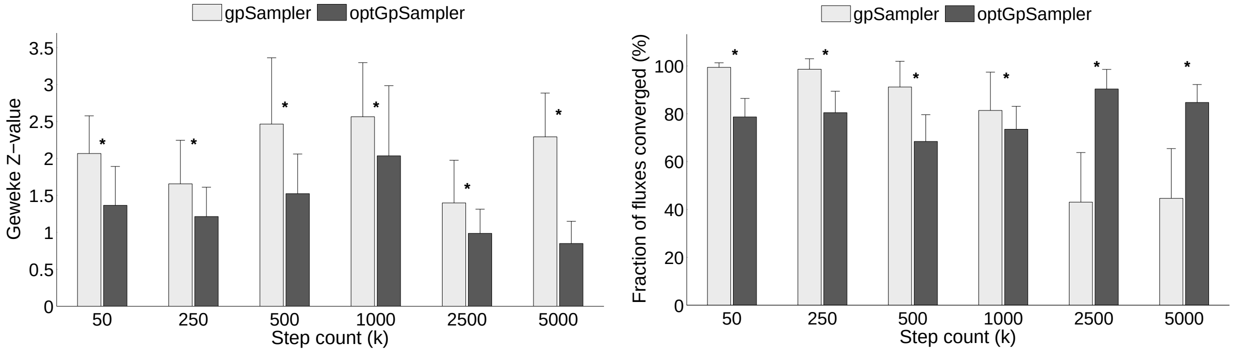

(a) Geweke diagnostic (b) Heidelberger-Welch diagnostic

Figure 3: **Empirical convergence for S. cerevisiae iND750.** Notice how *gpSampler* performs worse for the Geweke test when k increases. For the HW test, this effect is even more pronounced. *OptGpSampler* shows a similar effect as *gpSampler* did in Fig. 3 of the main text: performance drops initially and goes up again at high step counts.

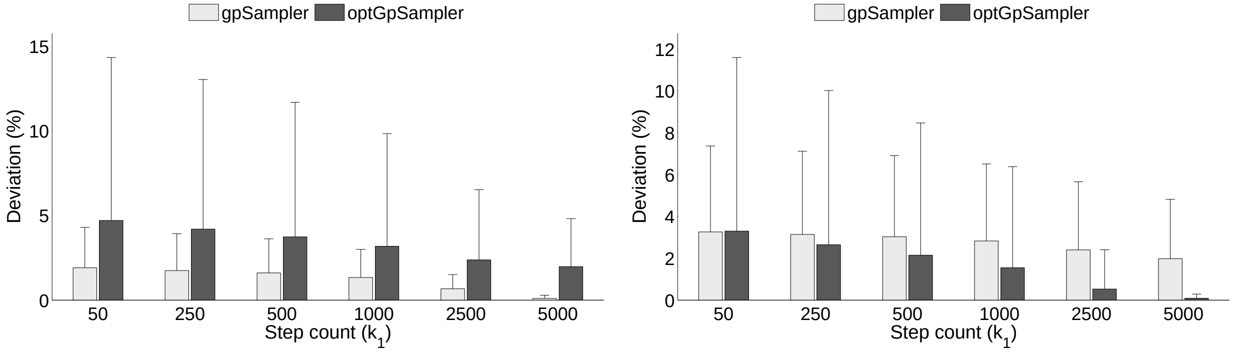

(a) y=gpSampler (b) y=optGpSampler

Figure 4: xy**-deviation for S. cerevisiae iND750.** xy-deviation of sampler x using step count k_1 to sampler y using k_2=5000. (a) y=*gpSampler*. (b) y=*optGpSampler*.
Results are in accordance with the empirical convergence results above. Cross-deviation remains large, even at high step counts. *OptGpSampler*'s deviation becomes significantly smaller around k=2500. At this step count, convergence results (see Fig. 3) also improve significantly.

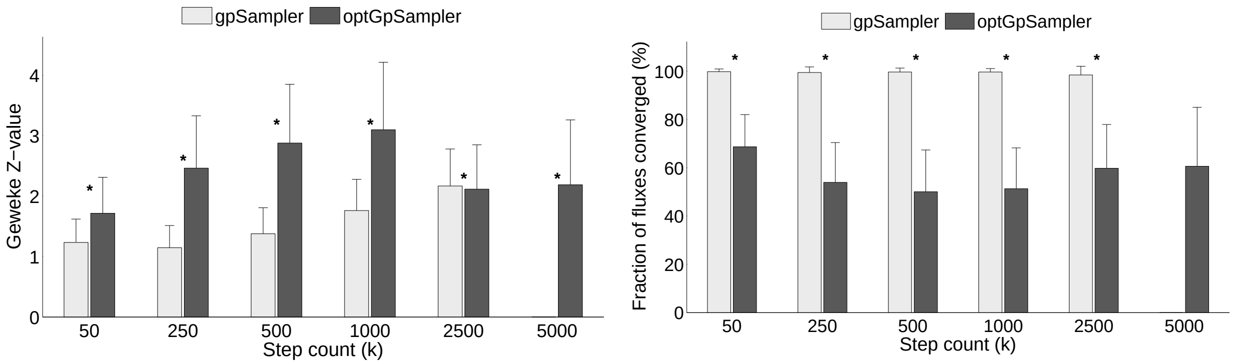

(a) Geweke diagnostic

(b) Heidelberger-Welch diagnostic

Figure 5: **Empirical convergence for H. sapiens recon1.** For this large network, the good convergence results for *gpSampler* may indicate convergence towards regions near the warm-up points. For *optGpSampler* convergence behaviour is similar as we saw for *gpSampler* on the previous models. Clearly, a large step count is required to sample this model.

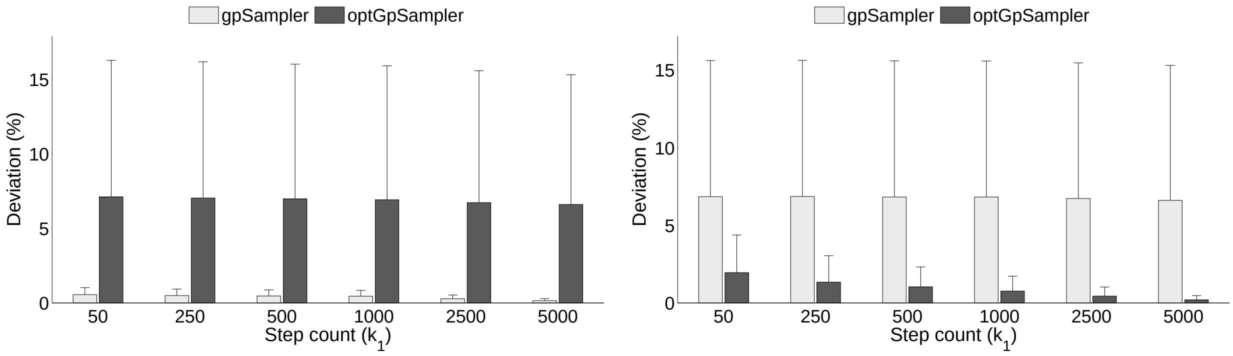

(a) y=gpSampler

(b) y=optGpSampler

Figure 6: xy**-deviation for H. sapiens recon1.** xy-deviation of sampler x using step count k_1 to sampler y using k_2=5000. (a) y=*gpSampler*. (b) y=*optGpSampler*.
Again, the samplers show a large cross-deviation ($x \neq y$). Furthermore, *gpSampler* hardly improves when the step count increases. Given the large dimensionality of the solution space, this may indicate a non-uniform sample spread. *OptGpSampler* improves more when k increases, which could indicate that the samples spread more uniformly over the space.

3 Reactions with smallest and largest deviation

Here we illustrate how a small or large xy-deviation leads to a similar or highly dissimilar sample distribution. We choose to compare the distribution of samples obtained with *gpSampler* and *optGpSampler* using step count k_1=500 to those obtained with *optGpSampler* at step count k_2=5000.

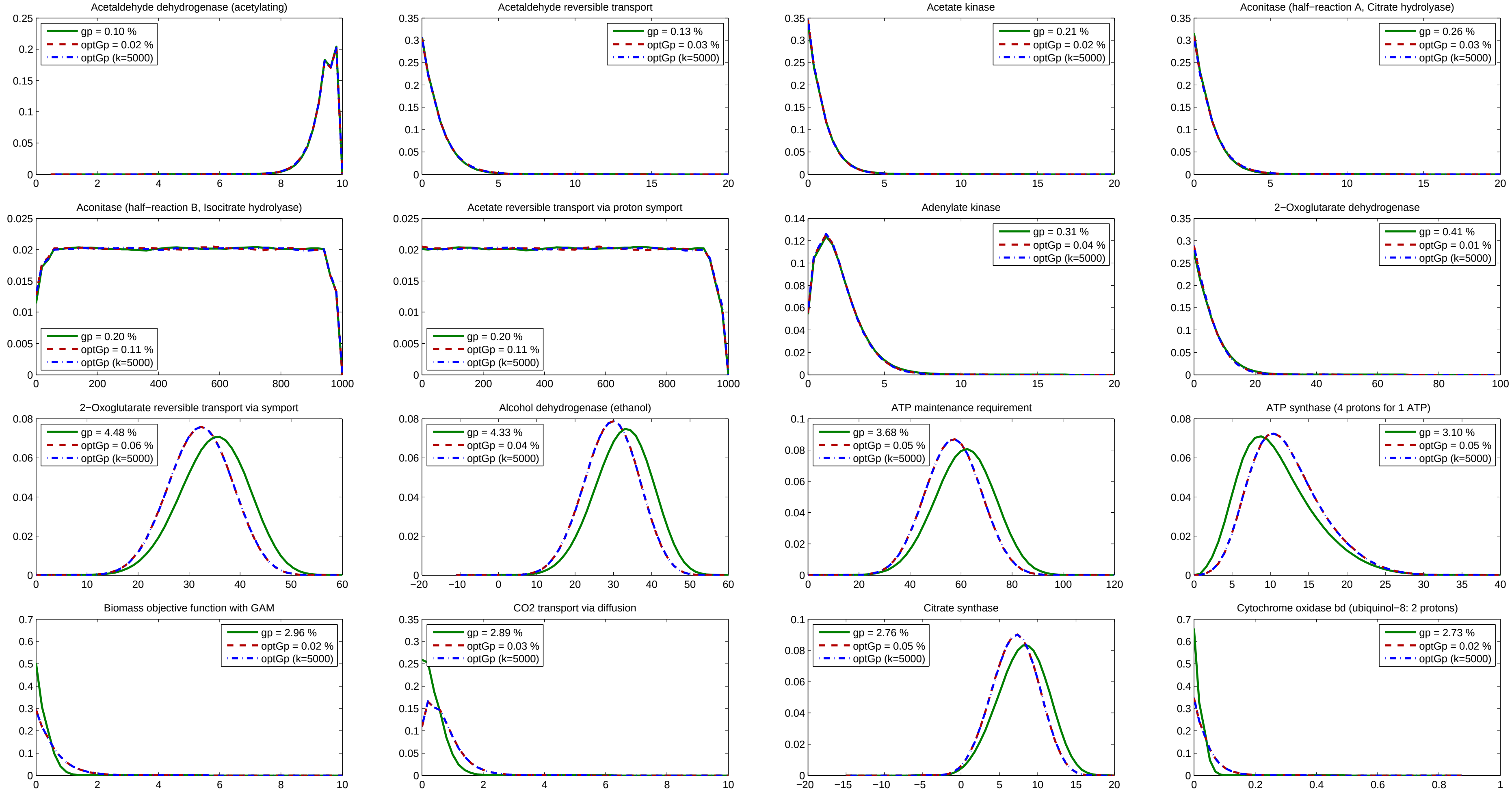

Figure 7: **E. coli central metabolism**
Here we show how a small or large xy-deviation affects the sample distribution. The fluxes with smallest average deviation (top 8) and largest average deviation (bottom 8) are shown. For this smallest network, the largest differences still give comparable distributions.

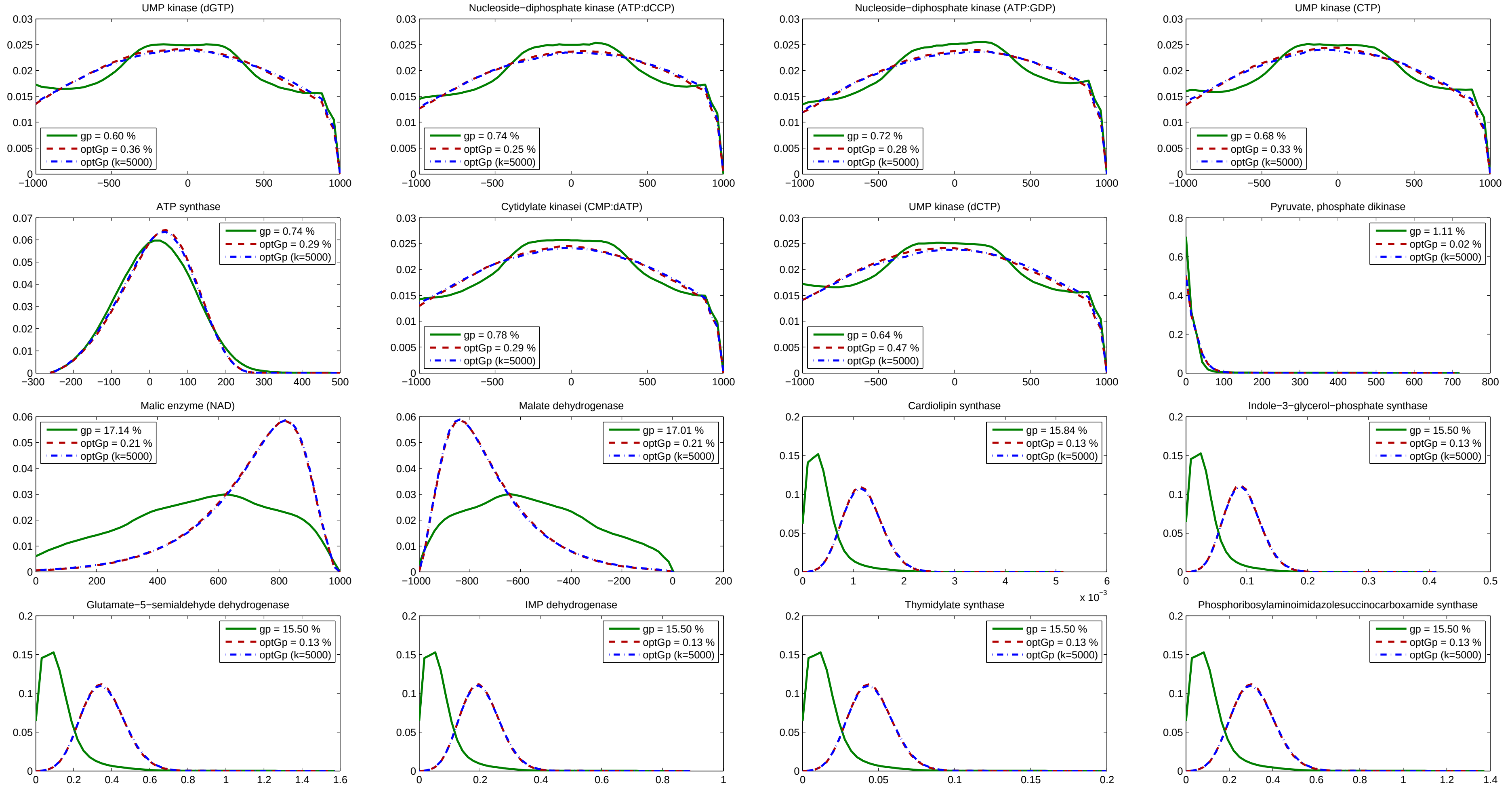

Figure 8: **C. thermocellum iSR432**

Here we show how a small or large xy-deviation affects the sample distribution. The fluxes with smallest average deviation (top 8) and largest average deviation (bottom 8) are shown. Notice that while the the sample distributions with smallest deviation are still similar, those with largest deviation are highly different.

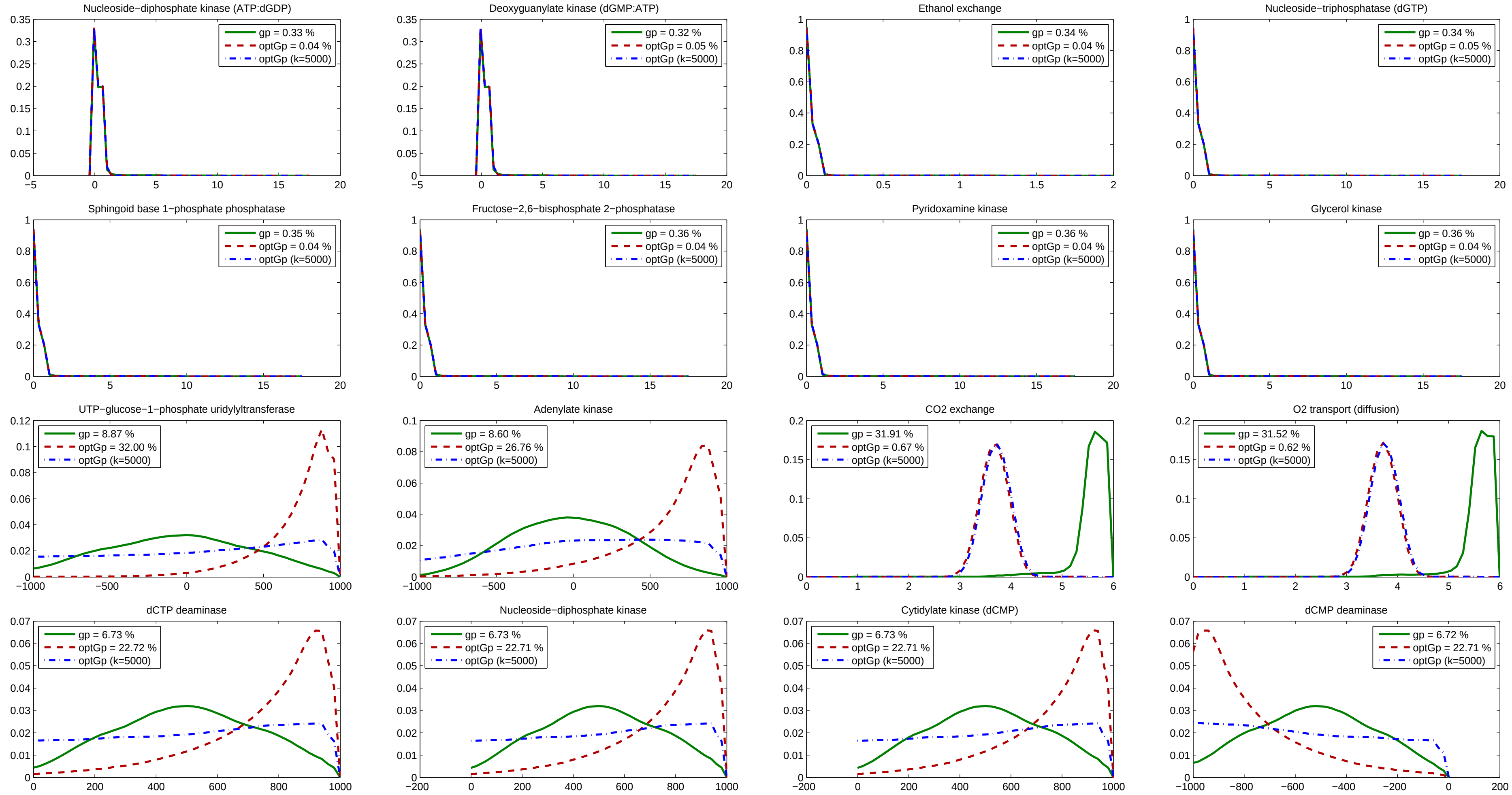

Figure 9: **S. cerevisiae iND750**

Here we show how a small or large xy-deviation affects the sample distribution. The fluxes with smallest average deviation (top 8) and largest average deviation (bottom 8) are shown. The smallest xy-deviation leads to almost identical distributions. The largest deviation gives completely different sample distributions.

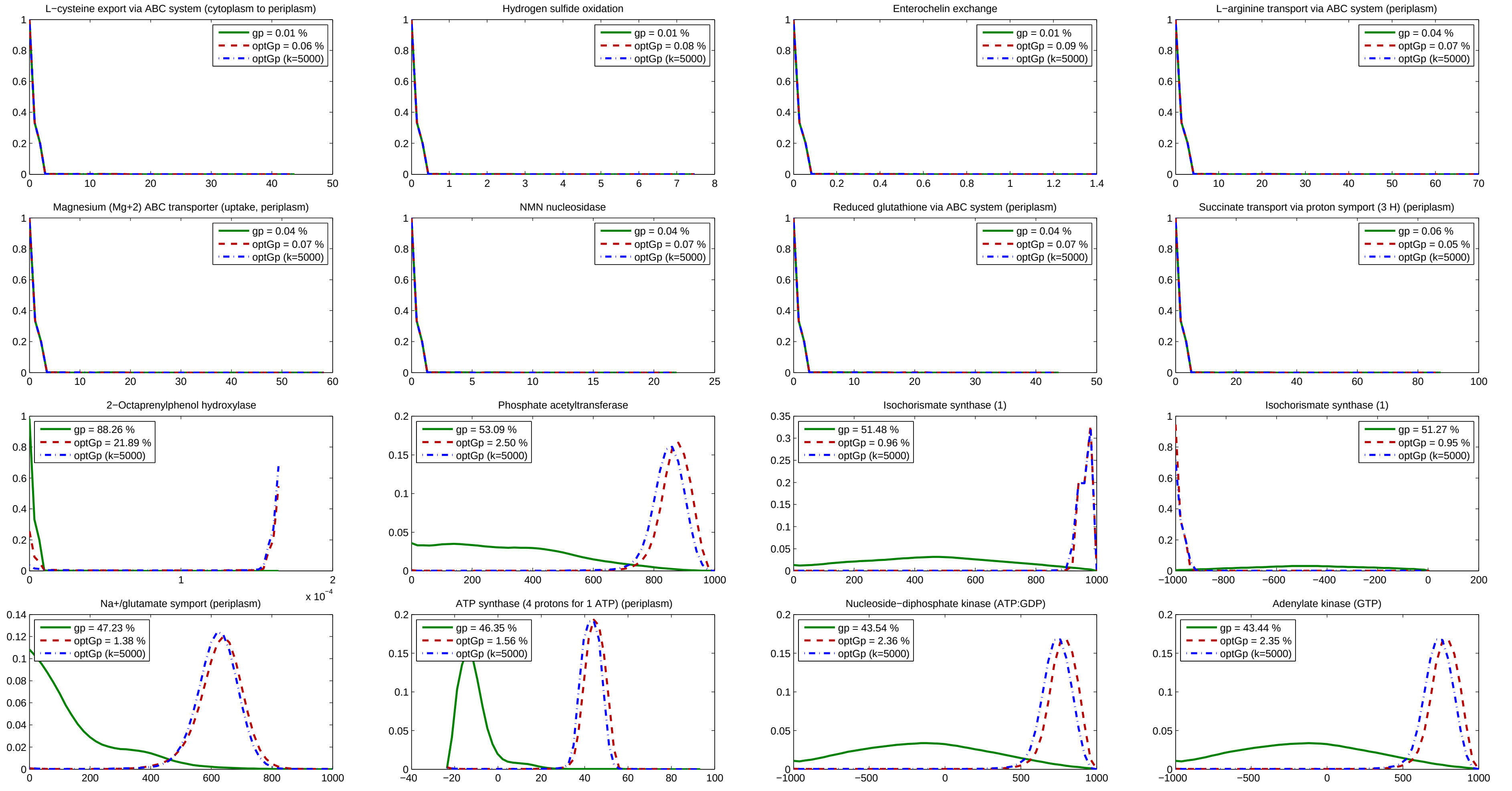

Figure 10: **E. coli iAF1260**

Here we show how a small or large xy-deviation affects the sample distribution. The fluxes with smallest average deviation (top 8) and largest average deviation (bottom 8) are shown. The smallest xy-deviation leads to almost identical distributions. The largest deviation gives completely different sample distributions.

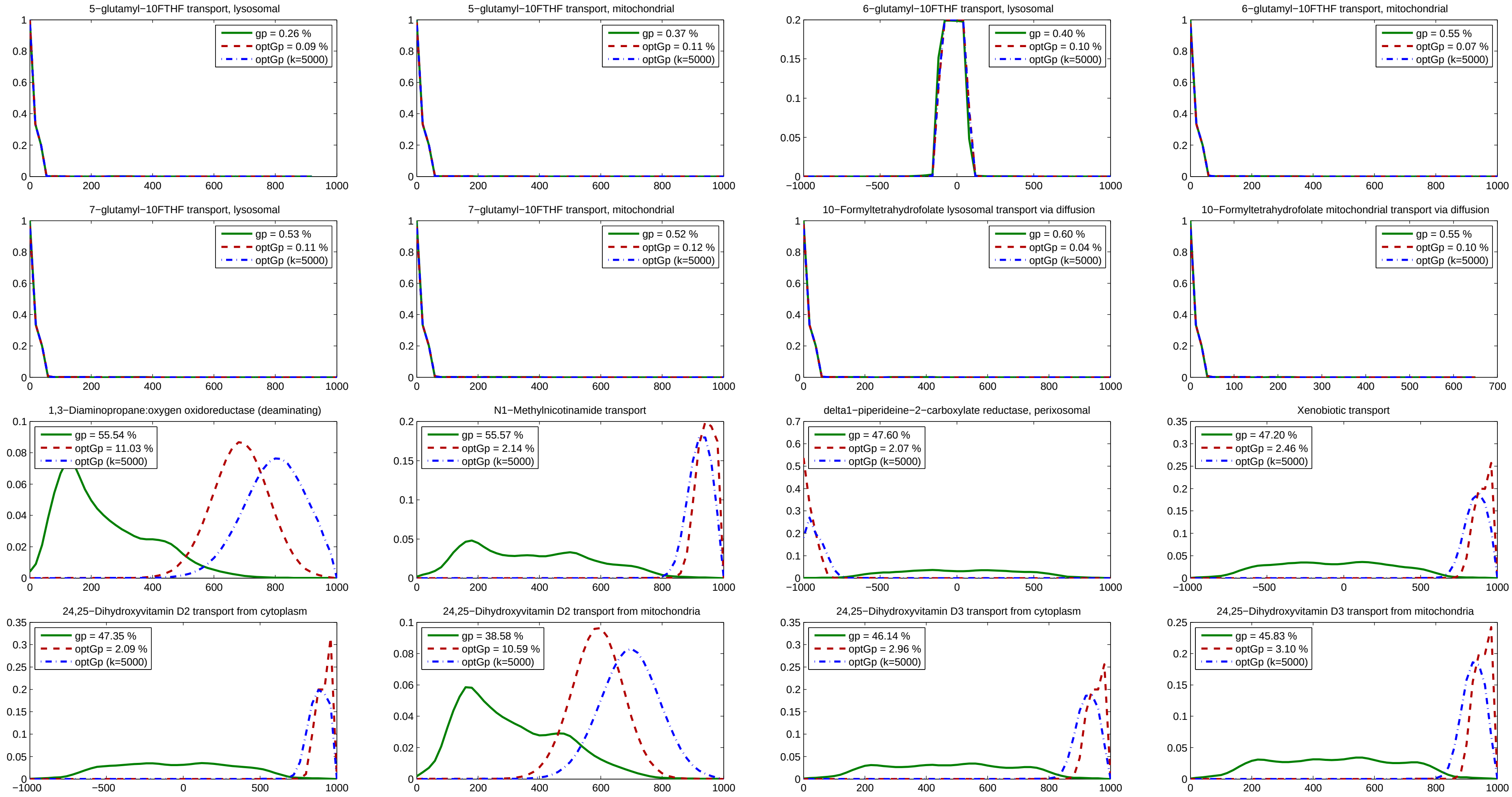

Figure 11: **H. sapiens recon 1**

Here we show how a small or large xy-deviation affects the sample distribution. The fluxes with smallest average deviation (top 8) and largest average deviation (bottom 8) are shown. The smallest xy-deviation leads to almost identical distributions. The largest deviation gives completely different sample distributions.
